# Gabapentin for Post-Hospitalization Alcohol Relapse Prevention; Should Gabapentin Be Considered for FDA Approval in the Treatment of Alcohol Use Disorder?: A Case Presentation and Literature Review

**DOI:** 10.7759/cureus.8931

**Published:** 2020-06-30

**Authors:** Ian H Rutkofsky, Kristy A Fisher, Clara L Alvarez Villalba, Samuel Neuhut

**Affiliations:** 1 Psychiatry, Aventura Hospital and Medical Center, Aventura, USA

**Keywords:** alcohol use disorder, naltrexone, acamprosate, disulfiram, gabapentin

## Abstract

Alcohol use disorder (AUD), a chronic condition that affects many people worldwide, is characterized most commonly by a preoccupation with alcohol, an irresistible craving for or the inability to control the consumption of alcohol, and the marked resultant disturbance it bestows upon one’s life. Although a difficult and time-consuming condition to attempt to treat, there are currently three FDA-approved medications for AUD, including naltrexone, acamprosate, and disulfiram. However, literature points towards another agent, gabapentin, that may be efficacious in preventing relapse symptoms and cravings with enhanced effectivity in reducing post-hospitalization alcohol consumption behaviors. In this paper, we discuss a case presentation and literature review demonstrating the role of gabapentin in treating AUD and symptoms associated with alcohol withdrawal, along with its potential use in relapse prevention.

## Introduction

Alcohol use disorder (AUD) is a chronic condition often characterized as a relapsing brain disease involving compulsive alcohol use, with loss of control over the cravings and resultant consumption of alcohol. Currently, there is an estimated 30 million people who meet the diagnostic criteria for AUD defined in the Diagnostic and Statistical Manual of Mental Disorders, 5th edition (DSM-5) [1]. Currently, there are three FDA-approved medications for the treatment and management of AUD: naltrexone, acamprosate, and disulfiram. While these medications have proven to be efficacious for a large number of patients, there still remains a significant population size that indicates inadequacy in the prevention of relapse with alcohol with the use of these agents. These inadequacies are likely due to the limited targets of treatment for each agent in the management of AUD [2]. Literature, including data collected from systematic reviews, by Ahmed et al., and a meta-analysis, by Kranzler et al., support the evidence that gabapentin is effective for AUD in preventing relapse symptoms and cravings associated with alcohol cessation [3-4]. Gabapentin has also shown efficacy in reducing post-hospitalization alcohol drinking behaviors [5]. In this paper, we discuss a case presentation and literature review demonstrating the role of gabapentin in treating AUD and symptoms associated with alcohol withdrawal, along with its potential use in relapse prevention.

## Case presentation

The patient is a 36-year-old divorced, white female, with reported past medical history of hepatitis C and psychiatric history of post-traumatic stress disorder (PTSD), major depressive disorder (MDD), AUD, cocaine use disorder, and cannabis use disorder, who presented voluntary to our inpatient psychiatric facility with depressed mood and suicidal ideation. The patient reported starting gabapentin approximately six months prior and had since maintained sobriety until recent events leading up to her admission: medication noncompliance for eight weeks with progressive alcohol cravings and relapse of 6-12 beers daily and varying amounts of vodka for the past two weeks. The patient also reported a relapse on cocaine while intoxicated three days prior to hospitalization. Last alcoholic consumption was approximately 24 hours prior to admission.

Physical exam

General Assessment

The patient was admitted to our acute psychiatric inpatient facility. Upon initial assessment, the patient appeared alert and oriented, with flat affect and depressed mood. The patient denied auditory or visual hallucinations, delusions, paranoia, or symptoms of mania. The patient’s chief compliant was intense feelings of anxiety, associated with alcohol cravings. On evaluation, vital signs were: HR: 78; RR: 18; BP: 131/63; 02 Sat.: 99% on room air. Labs including complete blood count (CBC) and comprehensive metabolic panel (CMP) were unremarkable. Urine drug screen was positive for cocaine and cannabinoids and blood alcohol level was undetectable.

Plan

The patient was started on fluoxetine 10 mg PO, for mood, gabapentin 400 mg BID PO for anxiety, alcohol cravings and anxiety. The patient was started on the CIWA protocol with lorazepam 1 mg PO PRN x 1 day, for monitoring and withdrawal symptoms along with folic acid 1 mg/day, thiamine 100 mg/day, and multivitamins, for supplementation. Lorazepam 1 mg PO was given once in the ED. No lorazepam or librium taper was administered while on the inpatient unit. On day 3 of hospitalization, gabapentin was up-titrated to 600 mg BID PO daily, for better control of anxiety and alcohol cravings prior to her discharge.

Post-discharge status/follow-up

After discharge, the patient reported compliance with gabapentin 600 mg PO BID for approximately 45 days post-discharge. She subsequently relapsed on alcohol and cocaine one month following medication non-compliance, requiring readmission. In addition to medication non-compliance, the patient reported new stressors revolving around family dynamics, contributing to depression with suicidal ideations and resultant relapse. The patient was restarted on her medication regimen with an adjustment in antidepressants. Citalopram 20 mg PO daily was initiated in place of fluoxetine for better control over the patient’s depression and to sustain improved control of her alcohol cravings. After the third day of hospitalization, the patient was discharged home. The patient has not required readmission to our hospital since this last discharge date, approximately six months ago.

## Discussion

Diagnosis of alcohol use disorder (AUD) and severity

The patient discussed in this case presentation met the diagnostic criteria of AUD and fulfilled eight positive findings over the duration of a 12-month period, thereby encompassing a severity category of severe.

**Figure 1:**
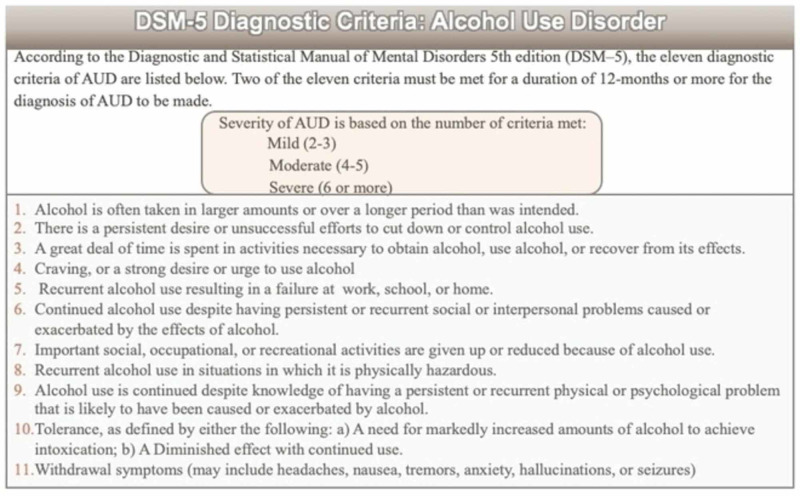
The diagnostic criteria of alcohol use disorder (AUD) according to the DSM-V. DSM-V: Diagnostic and Statistical Manual of Mental Disorders, 5th edition

Of note, literature reveals that gabapentin has shown efficacy in controlling alcohol cravings in all ranges of severity of AUD within the DSM-5. Eleven diagnostic criteria are listed in the DSM-5 in order to determine the appropriate diagnosis of AUD. Two of the eleven criteria must be met over a 12-month period to establish a diagnosis of AUD. The DSM-5 lists a range containing three degrees of severity, including mild, moderate, and severe, with severity being based on the number of diagnostic criteria met: mild (2-3), moderate (4-5), or severe (6 or more) (Figure 1).

FDA-approved medication for the prevention of alcohol relapse or as treatment for AUD

**Figure 2:**
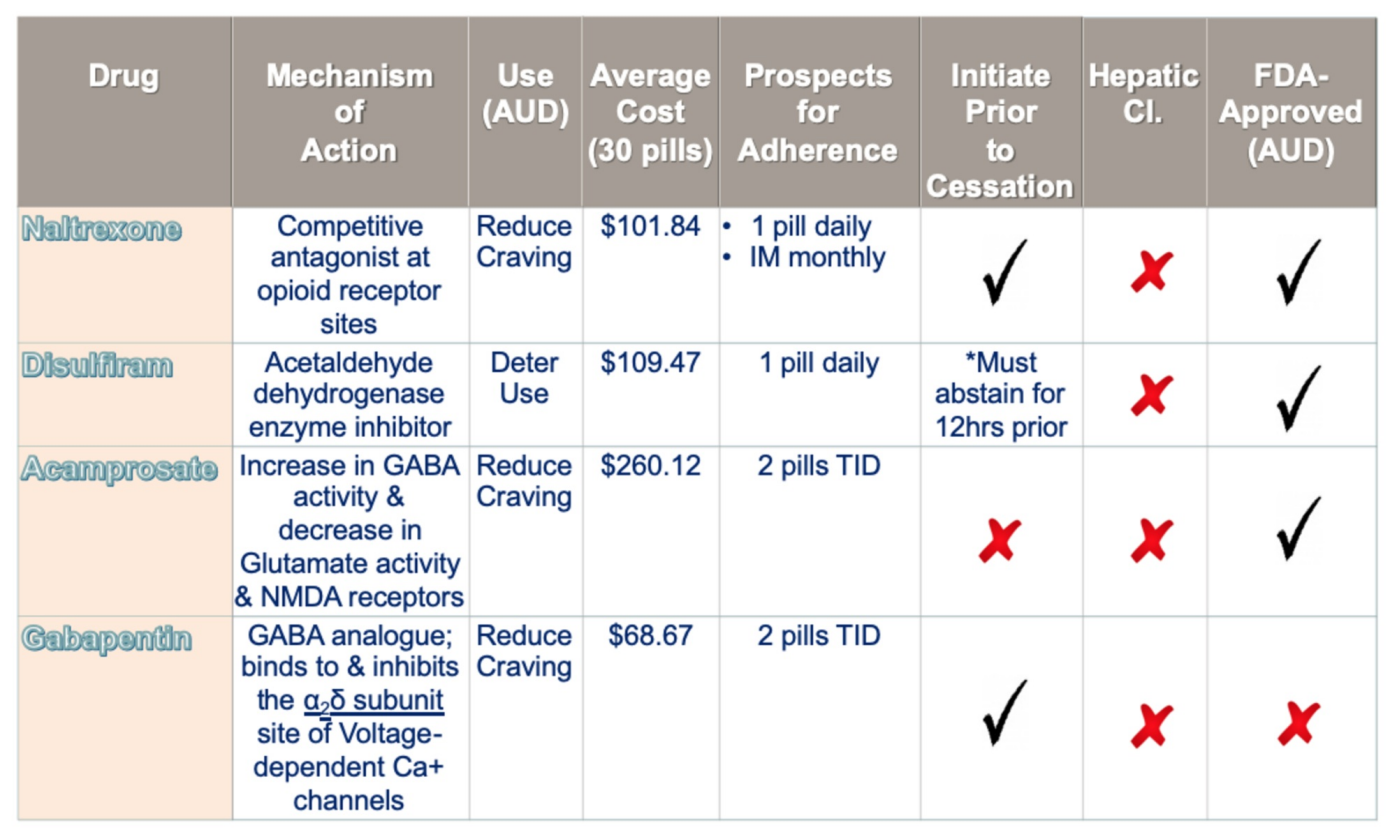
Medications commonly used in the management of alcohol use disorder (AUD). Comparison of gabapentin to three FDA-approved medications for the treatment of AUD, including naltrexone, acamprosate, and disulfiram.

Gabapentin is not currently recognized as an FDA-approved drug for the prevention of alcohol relapse or as treatment for AUD. However, literature clearly indicates its strong role in treating AUD and symptoms associated with alcohol cravings. Due to its efficacy, along with its lower cost and adverse effect profile, the authors of this paper feel it should be recognized as a potential agent in the prevention of relapse as either adjunctive or monotherapy in the treatment of AUD (Figure 2).

Off-label use of gabapentin in the treatment of AUD

Currently, there are only three medications approved for the treatment and management of AUD, including naltrexone, acamprosate, and disulfiram. Despite significant evidence throughout literature indicating efficacy of gabapentin in the management of AUD symptomatology and related cravings, it has remained an off-label recommendation for the management of AUD. None the less, naltrexone, acamprosate, disulfiram, and gabapentin are all agents commonly utilized by clinicians in the management of AUD throughout practice. As signified in Figure 2, these agents aid in the prevention of alcohol cravings. Furthermore, these agents have also shown to surpass any benefit to harm ratio, with fewer possible side effects of these anti-craving agents when compared to the actual associated harms of untreated AUD [6].

Throughout literature, many other agents have been tested for efficacy in the treatment and management of AUD. These other, off-label medications of interest include: topiramate, ondansetron, varenicline, baclofen, sodium oxybate, and selective serotonin reuptake inhibitors (SSRIs) [7]. Many different classes of antidepressants with various mechanisms of action have yielded mixed results. Conversely, acute serotonin reuptake inhibition has been shown to reduce cue-induced alcohol cravings, specifically with citalopram 40 mg daily [8].

Literature suggests that gabapentin, when titrated up to 1200 mg/d, orally, is conceivably the most efficacious treatment in patients with AUD with a history of more severe alcohol withdrawal symptoms [1]. Research suggests gabapentin may be approaching the brink of FDA-approval for alcohol cravings within the USA [7]. With anticipation, the authors of this paper believe gabapentin merits closer attention and approved regulations by the FDA as a first-line medication for alcohol cravings rather remaining its current status of off-label use.

Gabapentin combination therapy in the treatment of AUD

Naltrexone is a semi-synthetic opioid with competitive antagonist activity at the mu-opioid receptors. Naltrexone has demonstrated efficacy in the treatment of alcohol and opioid use disorders, and is currently recognized as a first-line treatment in AUD [9]. Naltrexone can currently be prescribed in two distinctive formulations, including daily oral tablet or via monthly injection. Daily use of naltrexone has been shown to reduce the likelihood of relapse drinking by 5% and binge-drinking by 10% [10]. Long-acting injectable (LAI) naltrexone differs from that of oral formulation because only need a single monthly injection is required, thus ultimately leading to increased compliance.

Literature demonstrates a longer median time to relapse for those treated with LAI naltrexone when compared to treatment via the oral formulation of naltrexone (150.5 days vs 50.5 days, P < .01) [11]. The pharmacokinetic differences of the LAI preparation when compared to that of the oral preparation may offer reasoning for the discrepancy in efficacy and tolerability for alcohol dependence. These findings warrant additional studies for further investigation [12].

However, naltrexone has not been shown to be universally efficacious alone, which may be due to the associated symptoms experienced during the phases of early abstinence, incorporating both insomnia, as well as mood instability [13]. Conversely, literature suggests the efficacy of gabapentin in the management of AUD, with abilities to provide both the reduction of these symptoms and the prevention of early relapse [3,5,13]. Recent clinical trials have indicated a longer interval to heavy drinking during the first six weeks of the abstinence period with patients taking naltrexone combined with gabapentin [13]. Such findings suggest not only gabapentin as a possible low-cost monotherapy, but also as adjunct with another currently FDA-approved medication as dual therapy for optimal symptomatology coverage and relapse prevention, thus providing a potentially superlative option in the management of AUD.

Furthermore, literature, along with the outcome of the case presentation, supports claims of other agents of various mechanisms of action as potential therapeutic adjuncts in the dual therapy of AUD with gabapentin. A recent controlled clinical trial on alcohol-dependent individuals taking citalopram 40 mg PO daily resulted in decreased cue-induced cravings for alcohol [8]. The patient in the case presentation was started on 20 mg of citalopram with combination therapy of gabapentin 600 mg PO BID daily for depression and alcohol cravings, respectively. We believe a clinical trial is justified for the combination therapy of citalopram 20-40 mg PO daily and gabapentin 600 mg PO BID daily for the management of alcohol cravings with any associated and/or causative major depression, along with post-hospitalization alcohol relapse prevention in the treatment of AUD.

Safety precautions in the use of anti-craving medication in the treatment of AUD

In 2004, acamprosate became the most recent medication to receive approval from the US FDA for the indication for AUD. Acamprosate, an analogue of I³-aminobutyric acid (GABA), is thought to reduce alcohol consumption by affecting calcium channels and modifying transmission of GABA and glutamate, thus ensuing decreased alcohol cravings. Acamprosate must be used with caution in the elderly patient population as well as in patients with comorbid acute renal failure, as it is contraindicated in patients with severe renal failure [14].

Naltrexone is a mu-opioid receptor antagonist and is understood to reduce the endogenous reward pathway signaling associated with ingestion of alcohol. Naltrexone has a black box warning for hepatocellular injury, but can be prescribed in patients with stable or compensated cirrhosis. Nevertheless, it is contraindicated in patients with acute hepatitis or liver failure [15]. Naltrexone may cause elevated liver enzymes, so liver function tests should be monitored periodically. Equality in safety outcomes were exhibited among the two formulations of naltrexone, including both oral and LAI [11]. Naltrexone should be avoided in patients currently using opioids as it may precipitate opiate withdrawal symptoms. However, naltrexone may be safely initiated on opiate-naive patients with comorbid opiate use disorder and AUD [16].

**Figure 3:**
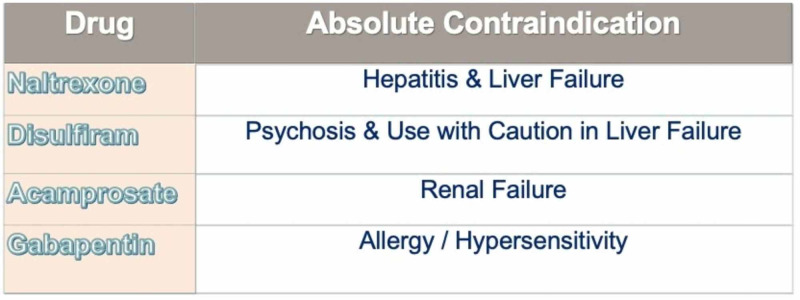
Contraindications in naltrexone, disulfiram, acamprosate and gabapentin.

Disulfiram is an FDA-approved treatment for AUD, but is often considered a second-line therapy after acamprosate and naltrexone. Disulfiram is contraindicated with recent use of metronidazole, severe coronary disease, heart failure, and psychosis. Side effects may include peripheral neuropathy, hepatitis, optic neuritis, depression, or worsening psychosis. Caution is required for patients with a history of liver disease [17]. In contrast, due to its limited side effect profile, minimal drug-drug interactions, and extensive pharmacokinetic safety profile in renal impairment, physicians have been able to confidently and comfortably prescribe gabapentin, particularly in the populations of advanced age and/or higher risk (Figure 3) [18].

## Conclusions

This young woman, diagnosed with AUD, initially presented with alcohol relapse and severe use disorder after non-compliance to her home medication regimen, which included gabapentin. She presented with the classic features of AUD, including intense cravings for and anxiety over alcohol consumption. Her cravings and anxiety were significantly reduced after the re-initiation of gabapentin, and became notably more stable upon the up-titration of gabapentin from 400 mg PO BID to 600 mg PO BID. The patient’s overall status displayed improvement with the diminution of her alcohol cravings. In summary, for individuals suffering from AUD, it is crucial to treat the patient and monitor for withdrawal symptoms with the definitive goal of long-term management of their condition. In order to do so, patients will most likely require long-term therapy, which significantly contributes to a sustained outcome of sobriety. The literature reported shows reduced levels of anxiety and better control of alcohol cravings with daily use of gabapentin, along with relapses in alcohol use with non-compliance of daily gabapentin. Naltrexone, acamprosate, and disulfiram, although already indicated agents for the treatment of AUD, are not found to be universally efficacious, which is likely due to symptoms experienced during the early abstinence phase. Furthermore, gabapentin is a low-cost alternative to other FDA-approved medications indicated in the treatment of AUD and may provide control of the symptoms commonly associated with early abstinence than the other conventional medications already indicated for AUD. Our patient’s progress, with unrelenting response and efficacy to gabapentin alone, merits further consideration for the use of gabapentin in the substantial reduction of alcohol-drinking behaviors, as either a mono-therapy or in combination with other FDA-approved agents, in the treatment and management of AUD.
